# Belowground Root Competition Alters the Grass Seedling Establishment Response to Light by a Nitrogen Addition and Mowing Experiment in a Temperate Steppe

**DOI:** 10.3389/fpls.2022.801343

**Published:** 2022-07-14

**Authors:** Mingxing Zhong, Chun Liu, Xiukang Wang, Wei Hu, Ning Qiao, Hongquan Song, Ji Chen, Yuan Miao, Gang Wang, Dong Wang, Zhongling Yang

**Affiliations:** ^1^Tourism College, Xinyang Normal University, Xinyang, China; ^2^International Joint Research Laboratory of Global Change Ecology, School of Life Sciences, Henan University, Kaifeng, China; ^3^Department of Ecology, Jinan University, Guangzhou, China; ^4^College of Life Sciences, Yanan University, Yan'an, China; ^5^Key Laboratory of Mollisols Agroecology, Northeast Institute of Geography and Agroecology, Chinese Academy of Sciences, Harbin, China; ^6^College of Geography and Environmental Science, Henan University, Kaifeng, China; ^7^Department of Agroecology, Aarhus University, Tjele, Denmark; ^8^Laboratory of Resources and Applied Microbiology, School of Life Sciences, Henan University, Kaifeng, China

**Keywords:** belowground competition, land use change, light competition, nitrogen addition, seedling germination

## Abstract

Predicting species responses to climate change and land use practices requires understanding both the direct effects of environmental factors as well as the indirect effects mediated by changes in belowground and aboveground competition. Belowground root competition from surrounding vegetation and aboveground light competition are two important factors affecting seedling establishment. However, few studies have jointly examined the effect of belowground root and light competition on seedling establishment, especially under long-term nitrogen addition and mowing. Here, we examined how belowground root competition from surrounding vegetation and aboveground light competition affect seedling establishment within a long-term nitrogen addition and mowing experiment. Seedlings of two grasses (*Stipa krylovii* and *Cleistogenes squarrosa*) were grown with and without belowground root competition under control, nitrogen addition, and mowing treatments, and their growth characteristics were monitored. The seedlings of the two grasses achieved higher total biomass, height, mean shoot and root mass, but a lower root/shoot ratio in the absence than in the presence of belowground root competition. Nitrogen addition significantly decreased shoot biomass, root biomass, and the survival of the two grasses. Regression analyses revealed that the biomass of the two grass was strongly negatively correlated with net primary productivity under belowground root competition, but with the intercept photosynthetic active radiation in the absence of belowground root competition. This experiment demonstrates that belowground root competition can alter the grass seedling establishment response to light in a long-term nitrogen addition and mowing experiment.

## Graphical Abstract

Effects of light competition and belowground competition on the seedling of *S. krylovii* and *C. squarrosa* under the control, mowing, and N addition treatment. 
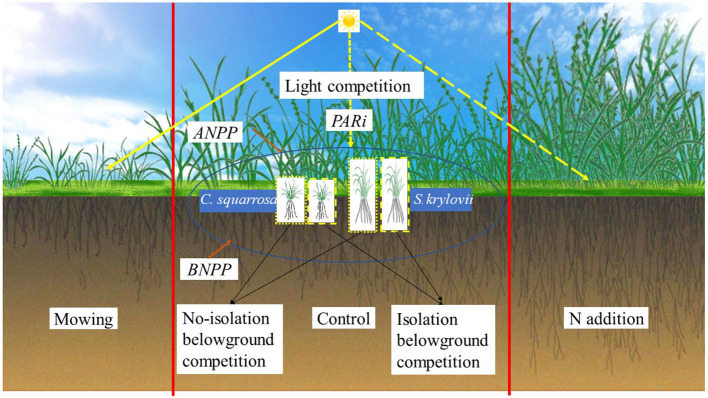

## Introduction

The seedling stage is a critical phase of plant growth that has a major effect on the structure and composition of natural communities (Ding et al., 2016; Peay and Clemmensen, 2018; Tomlinson et al., 2018; Zhou et al., 2021). Characterizing the responses of different plant seedlings to various factors affecting growth, survival, and biomass allocation can improve our understanding of community assembly and the mechanisms maintaining diversity in natural and disturbed ecosystems (Liu et al., 2012; Zhang et al., 2017a, 2020; Zhong et al., 2019). However, in recent years, relatively few experiments have been conducted to study the response of seedlings to global change, especially *in situ* experiments in the field.

Nitrogen (N) deposition is a major global driver of plant diversity loss that is predicted to increase in the future (Clark et al., 2007; Galloway et al., 2008; Bobbink et al., 2010; Liu et al., 2013b; Li et al., 2021). Most studies of nutrient-induced plant species loss have focused on competition-based mechanisms (Gilliam, 2006; DeMalach et al., 2017), including belowground competition and aboveground competition (light) (Ceulemans et al., 2017; Broadbent et al., 2018; Zheng et al., 2019). Among them, the effect of light competition on plant growth is a hot research topic in nutrient experiment in recent years (DeMalach et al., 2016, 2017; Xiao et al., 2021). Numerous studies have shown that light competition, with a lower light acquisition per unit biomass for small plants, has been proposed as a major mechanism of species loss after nutrient addition (DeMalach et al., 2016, 2017; Xiao et al., 2021). There are also studies showing that under nutrient addition, light is an important contributor affecting diversity replenishment, but not a decisive factor (Harpole et al., 2017). However, none of these studies discuss the role of belowground root competition. Belowground root competition is also an important factor affecting plant growth, especially in nutrient addition experiments (Träger et al., 2019; Wang et al., 2019). Differences in responses of large and small plants to belowground root competition may alter plant responses to light competition, affecting plant diversity. But how large and small plants respond to belowground root competition is unclear, especially in nutrient addition experiments.

Mowing for hay is a common land use type in grassland management that has a considerable effect on plant diversity and environment characteristic (Socher et al., 2012; Yang et al., 2012; Zhang et al., 2017b; DoleŽal et al., 2018; Huang et al., 2020). Mowing is often cited as an important mechanism for mitigating biodiversity loss from nutrient enrichment (Collins et al., 1998; Zhang et al., 2017b). Mowing can increase species richness by increasing light availability for small, subdominant plant species, thereby increasing germination rates and promoting seedling recruitment and plant growth (Collins et al., 1998; Yang et al., 2012; Stevens, 2016). Likewise, mowing alters belowground root competition, which in turn affects the establishment of seedlings of different plants. However, which competition is more important, we do not know.

Grasslands contain ~37% of the vegetation in terrestrial ecosystems and are one of the most important ecosystems in terms of their contribution to global food production (O'Mara, 2012; Wang et al., 2021a). Nutrient enrichment and mowing are two common management practices for increasing the use of grassland ecosystems (Humbert et al., 2016; Zhang et al., 2017b; DoleŽal et al., 2018), but their effect on seedling establishment remains unclear, especially in the nutrition experiment. Although most grassland species are perennials, seedling establishment is still an important factor affecting the structure of grassland plant communities. Here, we conducted a seedling transplant experiment within a multi-year N addition and mowing experiment, simulating seedling builds respond to aboveground light and belowground root competition. Our study species were *Stipa krylovii* (large plant) and *Cleistogenes squarrosa* (small plant), which are the most common grasses at the grassland study site. By studying the responses of plants of different sizes to aboveground and belowground competition in nutrient addition and mowing experiments, the mechanism of plant diversity loss under nutrient addition was explored.

## Materials and Methods

### Site Description and Species Selection

This experiment was conducted at Duolun Restoration Ecology Station, which is located in southern Inner Mongolia Autonomous Region (42°02'N, 116°17'E, 1,324 m a.s.l). The long-term (1954–2013) mean annual precipitation is 385 mm, and the mean annual temperature is 2.1°C. Ninety percent of the precipitation occurs between May and October. Monthly mean temperature ranges from −17.6°C in January to 19.2°C in July. The soil is classified as chestnut according to the Chinese classification. Dominant plant species in the temperature steppe include the perennial herbs *Stipa krylovii, Cleistogenes squarrosa*, and *Agropyron cristatum* (Yang et al., 2012).

In this study, the two most common grasses, *S. krylovii* and *C. squarrosa*, were selected as the research objects in the grasslands of Inner Mongolia. *Stipa krylovii* is a grass that tends to grow in clusters with large individual (high: 30–80 cm), which is advantageous under nutrient enrichment (Zhao et al., 2016). By contrast, *C. squarrosa* is a lower cluster grass with small individual (high: 10–30 cm), which makes it more prone to loss under nutrient enrichment. Moreover, with a fibrous root system, *C. squarrosa* is considered as a key species for sustainable grassland development (Liang et al., 2002).

### Experimental Design

Our experiment was nested within an existing long-term mowing and N addition experiment that began in 2012 (Wang et al., 2020, 2021b). Five 24 × 4 m blocks were arranged into one row and five columns. Each block was randomly assigned to four plots, each 4 × 4 m, with four treatments: (1) control (C), (2) mowing (M), (3) N addition (N, ambient plus 10 g N m^−2^ year^−1^, NH_4_NO_3_), and (4) combined M with N addition (MN, Wang et al., 2020). The subset of 15 plots of control, mowing, and N addition treatments were used in this experiment.

We collected the seeds of our two study species from a natural community in 2016. These seeds were sown in a seedbed in the field on May 10, 2017, and were carefully nursed for 20 d. Previous observations indicated that the roots of these two species were distributed in the top 15 cm of soil (Yang et al., 2011). Cylindrical root ingrowth containers were made from rigid plastic mesh (diameter = 7 cm, length = 15 cm, mesh = 4 × 4 mm square) (Chen et al., 2017). These root ingrowth containers contain two specifications, one is made of dense mesh (15 cm long, 5 cm width, 50 μm mesh) and the other is made of sparse mesh (15 cm long, 5 cm width, 2 mm mesh). Dense mesh can isolation root competition of surrounding plants for target plants, but sparse mesh cannot. On June 1, 2017, 20 root ingrowth containers were installed in each subset plot, including 10 dense mesh and 10 sparse mesh, and filled with *in situ* soil. Ten *S. krylovii* seedlings were placed in five root ingrowth containers with dense mesh and five root ingrowth containers with sparse mesh in each subset plot. Ten *C. squarrosa* seedlings were placed in the other half of root ingrowth containers in each subset plot. The in each subset plot were watered with 200 ml every day and dead seedlings were replaced for the first week. The seedlings were left to grow naturally until the end of September when they were sampled. The results under the C, M and N treatment were analyzed in this experiment.

### Measurements of the Microenvironment

Photosynthetic active radiation (PAR) on the ground was measured three times per month near the seedling within each plot using a Li-Cor Quantum Sensor (Li-Cor, Lincoln, NE, USA) on clear days. Two PAR values of the upper part of the canopy (PAR*u*) and the surface (PAR*s*) were measured at each site. Intercept photosynthetic active radiation (PAR*i*) was calculated using the following formula: PAR*i* = (PAR*u* – PAR*s*)/PAR*u*.

### Plant Sampling

On September 30, 2017, we recorded the number of surviving individuals and measured the maximum height of each plant. All seedling in each plot were then taken out from the ingrowth containers. Because of the short time of the experiment, all the roots were located inside the ingrowth containers. Each seedling was separated into shoots and roots. Roots were gently washed from the soil. All samples were oven-dried at 65°C for 48 h and weighed.

In the middle of August 2017, we harvested the biomass of surrounding vegetation at the peak aboveground plant biomass according to species in a 1 × 1 m square in each plot. Aboveground net primary productivity was estimated using standing biomass. Two 50-cm-deep holes were excavated with a soil auger (5-cm internal diameter) in each plot. Soil was sieved through a 2-mm screen, and roots were washed to measure the belowground net primary productivity. All samples were oven-dried at 65°C for 48 h and weighed. Net primary productivity (NPP) is equal to aboveground net primary productivity plus belowground net primary productivity.

### Statistical Analyses

We used three-way ANOVAs to assess the effects of species, root isolation, and management strategy on biomass production, height, survival, shoot, root, and shoot/root ratio. Duncan's multiple range test was used to compare differences between treatments. Regression analyses were used to assess the contributions of NPP and PAR*i* to seedling characteristics of the two species. All statistical analyses were performed in R 3.5.0 (Team, 2018).

## Results

### Individual Biomass, Height, and Survival

**Figure 1:**
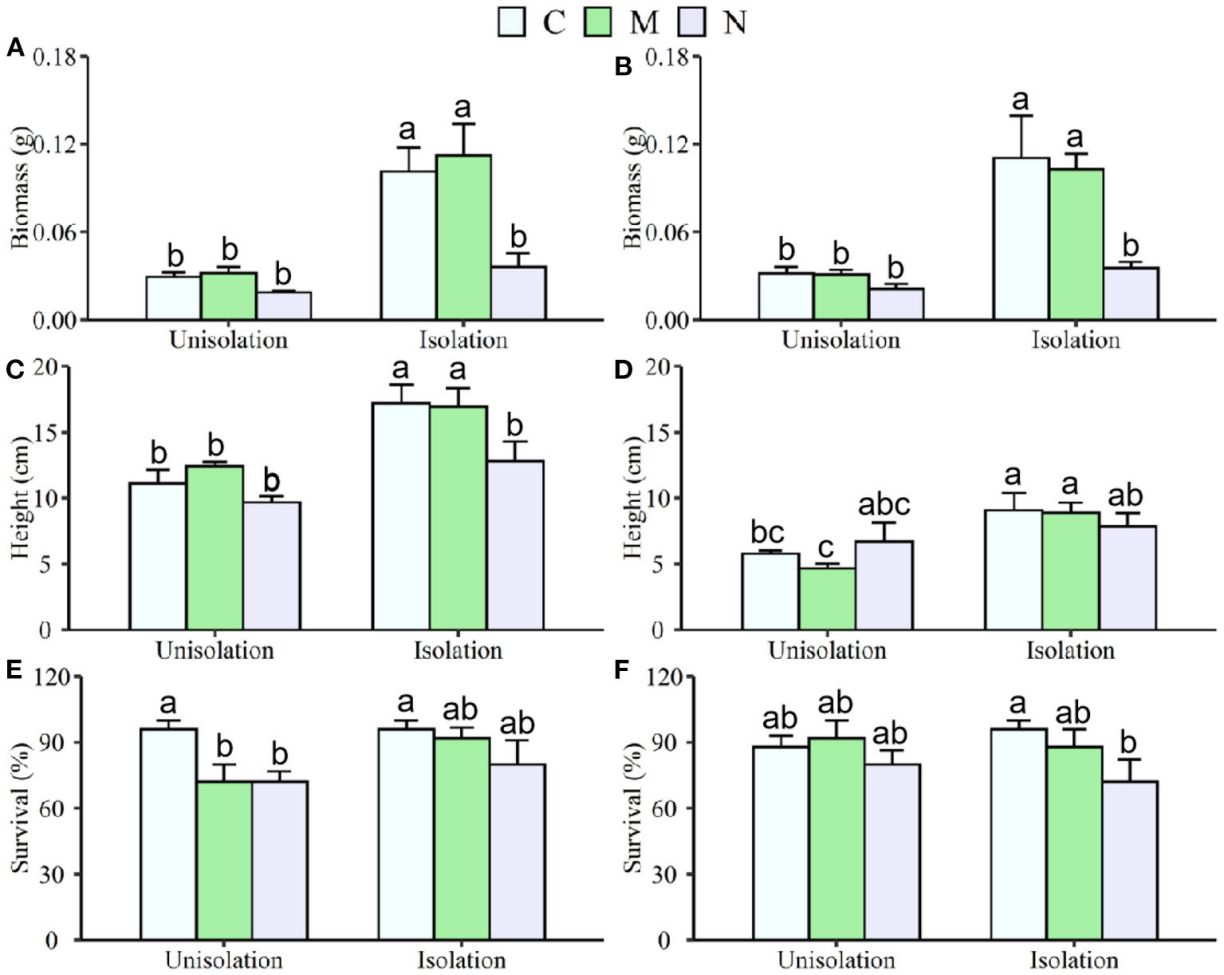
Effects of root isolation7 on biomass, height, and survival of *S. krylovii* **(A,C,E)** and *C. squarrosa* **(B,D,F)** in control (C), mowing (M), and N addition (N) plots. Different lowercases indicate significant differences among the three treatments at *p* < 0.05.

The belowground root isolation (RI) treatment significantly increased mean individual biomass and height by 207 and 46% (Table 1; Figure 1) across both *S. krylovii* and *C. squarrosa*, respectively. N addition significantly decreased mean individual biomass and survival by 60 and 18%, respectively (Supplementary Table S1; Figure 1). There was a significant interaction effect between RI and N on individual biomass (Supplementary Table S1). RI significantly increased the individual biomass of *S. krylovii* and *C. squarrosa* by 226 and 189% and their height by 47 and 50%, respectively (Table 2; Figure 1). N addition significantly decreased the individual biomass of *S. krylovii* and *C. squarrosa* by 57 and 63% and their survival by 20 and 15%, respectively (Supplementary Table S2; Figure 1). Mowing did not affect the individual biomass of *S. krylovii* and *C. squarrosa*. However, the survival of *S. krylovii* was reduced by 14% under the mowing treatment (Supplementary Table S2; Figure 1). There was a significant interaction between RI and N addition on the biomass of *S. krylovii* (Supplementary Table S2).

**Table 1:** Results (*F*-values) of three-way ANOVA on the effects of species (SP), root isolation (RI), management strategy (MS: control, N addition, mowing), and their interactions on total biomass, height, survival, shoot and root biomass, and root/shoot.

**Source of variation**	**Total biomass**	**Height**	**Survival**	**Shoot**	**Root**	**Root/shoot**
SP	0.00	103.354***	0.21	0.92	4.089*	1.06
RI	58.761***	36.372***	1.28	59.886***	44.29***	13.18**
MS	14.315***	2.76	6.169*	13.27***	13.154***	0.31
SP*RI	0.01	1.79	1.99	1.01	5.454*	17.116***
SP*MS	0.17	3.616*	0.65	0.43	1.39	3.311*
RI*MS	7.606**	1.66	0.33	7.496**	6.221**	0.90
SP*RI*MS	0.08	0.35	1.27	0.40	1.19	1.61

Significant level of F-value: *p < 0.05, **p < 0.01, ***p < 0.001.

**Table 2:** Results (*F*-values) of two-way ANOVA on the effects of root isolation (RI), management strategy (MS: control, N addition, mowing), and their interactions on total biomass, height, survival, shoot and root biomass, and root/shoot of *S. krylovii* and *C. squarrosa*, respectively.

**Source of variation**		**Total biomass**	**Height**	**Survival**	**Shoot**	**Root**	**Root/shoot**
*S. krylovii*	RI	31.515***	21.266***	3.333	36.926***	24.738***	0.002
	MS	0.385	0.707	6.533*	0.387	0.372	2.107
	RI*MS	0.034	1.14	3.333	0.052	0.017	1.134
*C. squarrosa*	RI	24.944***	12**	0.004	25.399***	20.612***	21.153***
	MS	6.926**	0.282	2.641	6.594**	7.685**	2.152
	RI*MS	3.342	1.22	0.882	3.615*	2.239	1.152

Significant level of F-value: *p < 0.05, **p < 0.01, ***p < 0.001.

### Shoot and Root Mass and Root/Shoot Ratio

**Figure 2:**
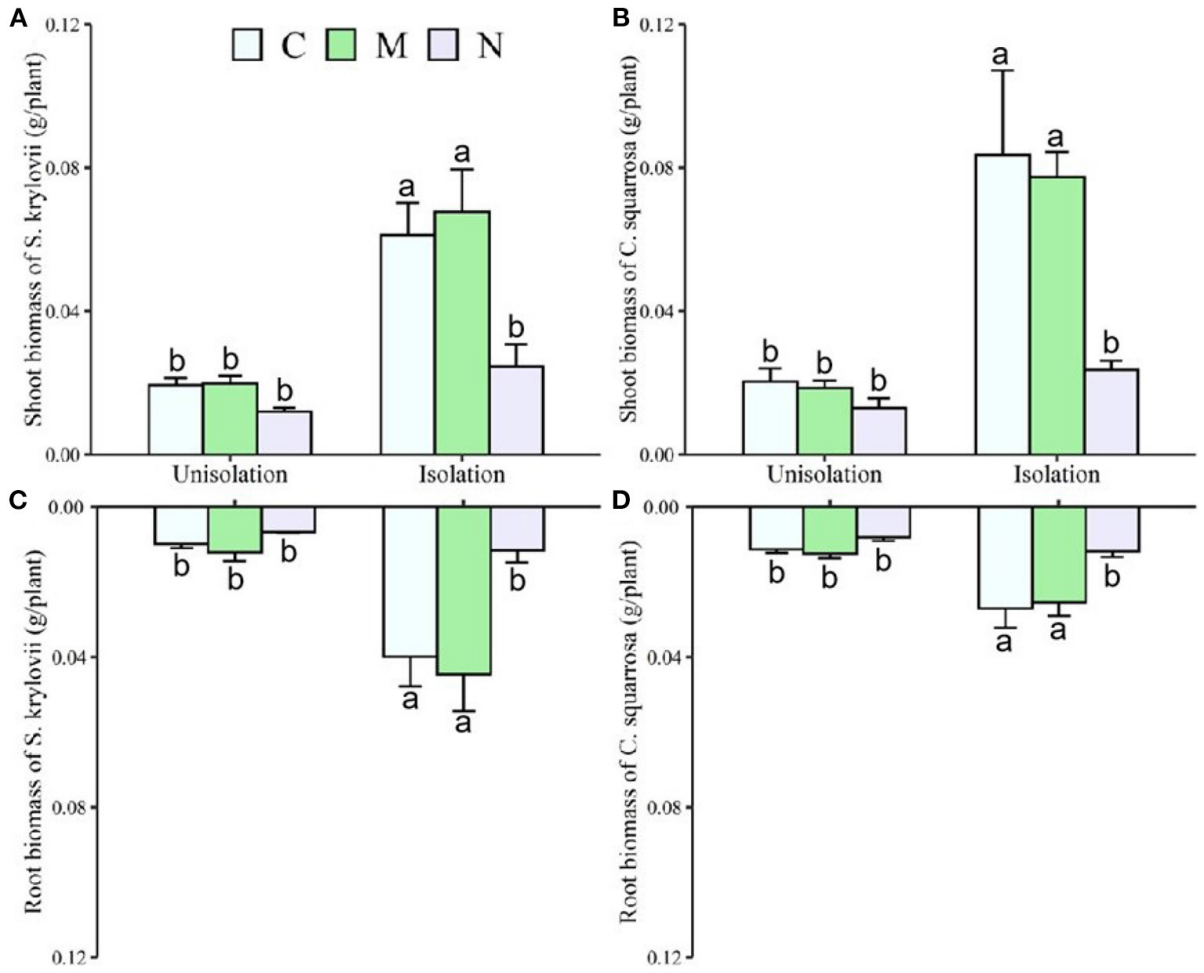
Effects of root isolation on shoot and root biomass of *S. krylovii* **(A,C)** and *C. squarrosa* **(B,D)** in control (C), mowing (M), and N addition (N) plots. Different lowercases indicate significant differences among the three treatments at *p* < 0.05.

**Figure 3:**
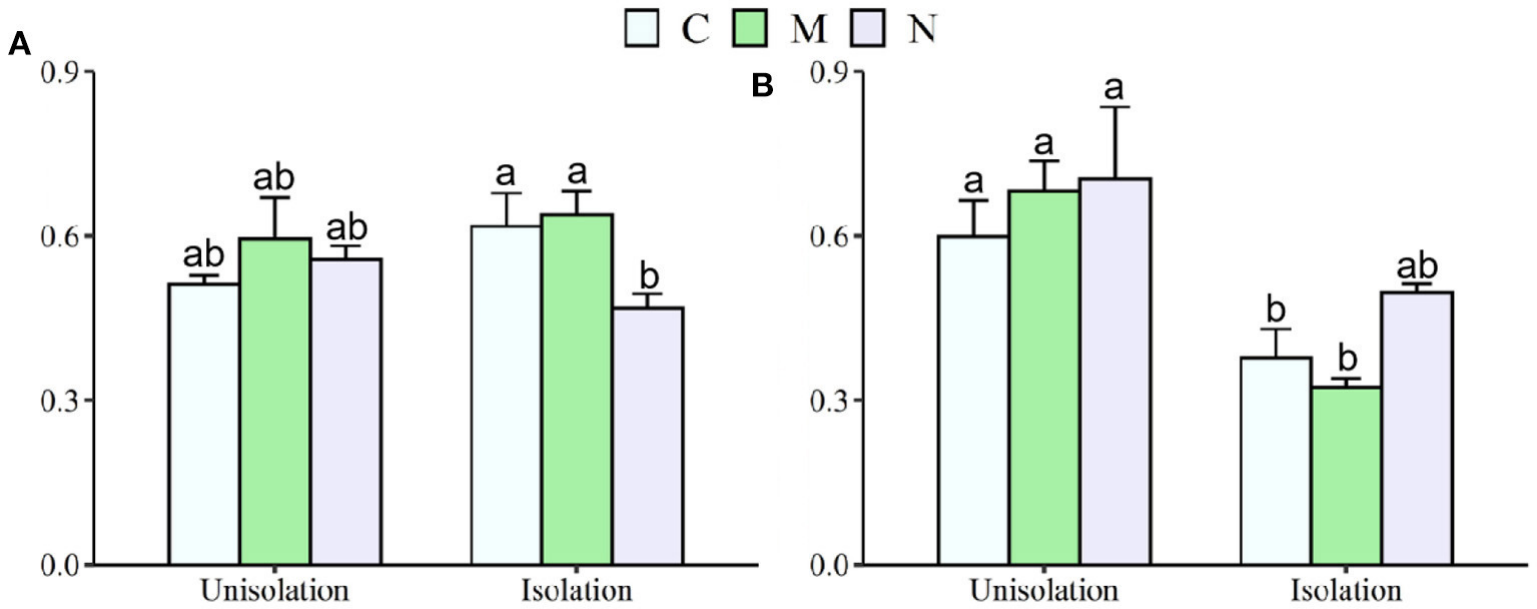
Effects of root isolation on root/shoot rate of *S. krylovii* **(A)** and *C. squarrosa* **(B)** in Control (C), mowing (M), and N addition (N) plots. Different lowercases indicate significant differences among the three treatments at *p* < 0.05.

Across the two species, RI treatment significantly increased mean shoot and root mass by 231 and 168%, respectively, and reduced the root/shoot ratio by 0.12 (Table 1; Figures 2, 3). N addition significantly decreased mean shoot and root mass by 61 and 58%, respectively (Supplementary Table S1; Figure 2). There was a significant interaction effect between RI and N on shoot and root mass (Supplementary Table S1). RI significantly enhanced the shoot mass of *S. krylovii* and *C. squarrosa* by 216 and 224% and root mass by 249 and 98%, respectively (Supplementary Table S2; Figure 2). N addition significantly decreased the shoot mass of *S. krylovii* and *C. squarrosa* by 54 and 68% and root mass by 63 and 52%, respectively (Supplementary Table S2; Figure 2). RI significantly decreased the root/shoot ratio of *C. squarrosa* by 0.25 (Table 2; Figure 3). N addition significantly increased the root/shoot ratio of *C. squarrosa* by 0.15 (Supplementary Table S2; Figure 3). There were significant interaction effects between RI and N on the shoot and root mass of *S. krylovii* and between RI and M on the shoot/root ratio of *C. squarrosa* (Supplementary Table S2).

### Relationships of Plant Performance With NPP and PARi

**Figure 4:**
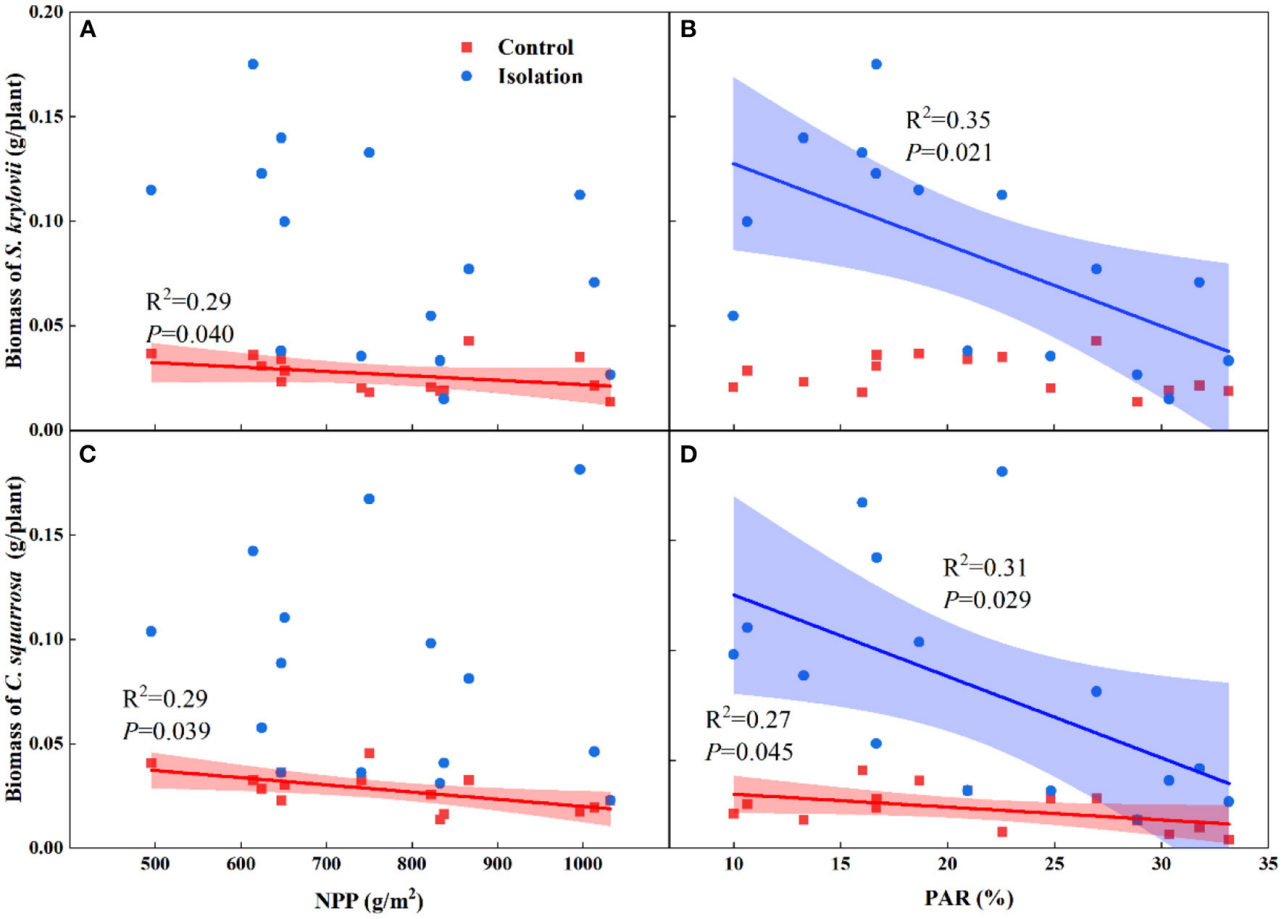
Relationships of total biomass of *S. krylovii* **(A,B)** and *C. squarrosa* **(C,D)** with net primary productivity (NPP) and lower canopy intercept photosynthetic active radiation (PAR*i*). Each data point represents mean biomass of each specie in each plot.

Simple linear regression analyses showed that the biomass of *S. krylovii* was negatively correlated with NPP under the no-isolation treatments (*R*^2^ = 0.29, *P* = 0.040, Figure 4A). The biomass of *S. krylovii* was negatively correlated with PARi under the isolation treatments (*R*^2^ = 0.35, *P* = 0.021, Figure 4B). The biomass of *C. squarrosa* was negatively correlated with NPP (*R*^2^ = 0.29, *P* = 0.039, Figure 4C) and PARi (*R*^2^ = 0.27, *P* = 0.045, Figure 4D) under the no-isolation treatments but was only negatively correlated with PARi under the isolation treatments (*R*^2^ = 0.31, *P* = 0.029, Figure 4D).

## Discussion

### Effects of N Addition and Mowing on Seedling Biomass

Nitrogen addition significantly decreased the individual seedling biomass of the two species, whereas mowing did not affect the biomass of these species. The negative responses of seedling biomass of the two species can be explained by indirect factors. Planting experiments showed that N addition increased biomass of seedling because of higher soil N availability (Ceulemans et al., 2017; Luo et al., 2020). But, these studies did not competition from surrounding plants. We found that N addition increased NPP, which was consistent with other studies conducted *in situ* ecosystems (DeMalach et al., 2017; Wang et al., 2017; Zhao et al., 2018). Therefore, N addition inhibits the growth of seedlings by increasing competition from surrounding vegetation (Jensen and Löf, 2017). On the one hand, N addition increase the height of the surrounding vegetation, increasing light competition (DeMalach et al., 2017). On the other hand, N addition can also increase the belowground root competition (Wang et al., 2019). Therefore, both aboveground light and belowground root competition combines to reduce biomass of seedling. Mowing can have a positive effect on seedling establishment (Bissels et al., 2006; Gibson et al., 2011). In some cases, mowing can decrease vegetation cover or NPP and increase ground light intensity (Collins et al., 1998; Gibson et al., 2011). However, we did not find a significant effect of mowing on the seedling biomass of the two species. This may stem from that the frequency of mowing was only once a year, which thus did not affect NPP and PAR*i*.

### Effects of N Addition and Mowing on Seedling Survival

Nitrogen addition and mowing decreased seedling survival in this experiment. Our results were inconsistent with previous studies on herbs (Jutila and Grace, 2002; Bissels et al., 2006; Zhang et al., 2018) or woody plants (Walters and Reich, 2000). This inconsistency might be explained by the different approaches used. Many previous studies have conducted planting experiments in greenhouses or fields in which the surrounding vegetation was absent (Walters and Reich, 2000; Zhang et al., 2018). However, the plots in our study were nested within a long-term N addition and mowing experiment. Both root and light competition are important factors that affect seedling survival (Gunaratne et al., 2011; Tomlinson et al., 2018; Hu and Wan, 2019). For example, nutrient enrichment can decrease seedling establishment in grassland by enhancing light asymmetry and interspecific competition (Xia and Wan, 2013; DeMalach et al., 2017). N enrichment can also increase the availability of toxic metals, which decreases seedling survival (Bobbink et al., 2010). Mowing can increase seedling establishment by removing the most productive plants and decreasing light competition (Collins et al., 1998; Gibson et al., 2011). But the decrease in seedling establishment due to mowing observed in this study may be caused by the lower soil nutrient content and soil quality after long-term clipping (Wang et al., 2020).

### Belowground Root Competition on the Seedling Characteristic

In our study, the performance of seedlings significantly increased in the RI treatment. These findings are consistent with previous studies showing that a low level of belowground root competition can maximize the success of seedling recruitment (Haugland and Tawfiq, 2001; Liu et al., 2013a). McConnaughay and Bazzaz (1991) suggested that root competition in the soil not only depletes water and nutrient but also creates physical barriers to root growth. The isolation of neighboring roots may, therefore, increase the physical space available for the growth of target seedling roots as well as reduce competition for other resources (Liu et al., 2013a). However, the effects of neighboring interactions on community structure differ at different phases of population growth. For example, competition associated with neighbors can accelerate seedling emergence (Dyer et al., 2008) but decrease seedling survival and biomass (Fayolle et al., 2009).

### Belowground Root Competition Alters the Relationship Between Light and Seedling Establishment

We used a simple correlation analysis to assess the relationship between seedling biomass and environmental factors. The negative relationships between seedling biomass and NPP are consistent with the results of many theoretical and empirical studies under the no-isolation treatments (Liu et al., 2007, 2013a). However, interspecific interactions can be complex (Martorell et al., 2014). Negative interspecific competition can occur when one species occupies the space required for another species to establish (e.g., mats of vegetation), and positive interactions can occur when, for example, adult plants create an optimal microclimate that facilitates the recruitment of small seeds and seedlings (Martorell et al., 2014). In our study, most species share similar ecological niches, so the relationship between species is more competitive than mutually reinforcing. Root and light competition are considered two important aspects of interspecific competition. Previous studies show that light competition is one of the main factors affecting seedling growth (Liu and Han, 2007; Fayolle et al., 2009; Liu et al., 2013a). However, other studies show that belowground root competition has been found to be more important than light competition in grasslands (Cook and Ratcliff, 1984; Haugland and Tawfiq, 2001). In our study, light competition becomes an important factor affecting seedling biomass when belowground root competition is isolation (Figure 4). Further analysis of previous studies found that the experiments that considered light competition as the main factor were mostly greenhouse experiments or planting experiments (Hautier et al., 2009), while the experiments that considered belowground root competition to be the main factor were mostly *in situ* experiments (Cook and Ratcliff, 1984; Haugland and Tawfiq, 2001; Wang et al., 2021c). Therefore, our study suggests that belowground root competition alters the relationship between light and seedling establishment (Graphical Abstract).

## Conclusions

Nitrogen addition significantly decreased the biomass and survival of seedlings. Grass seedlings achieved higher biomass and height under belowground root competition. NPP was negatively related to biomass under belowground root competition. However, the intercept PAR significantly affected the biomass of the two grass species in the absence of belowground root competition. The differential effects of belowground root competition and management strategy on seedling characteristics were largely attributed to the indirect effects of changes in NPP and light. Our findings provide insight into the mechanisms underlying the response of seedlings to aboveground and belowground root competition, information that is crucial for predicting the responses of species to global change.

## Data Availability Statement

The original contributions presented in the study are included in the article/Supplementary Material, further inquiries can be directed to the corresponding authors.

## Author Contributions

ZY designed the research. MZ, DW, and ZY collected data and performed the analysis. All authors wrote the article, contributed critically to the drafts, and gave final approval for publication.

## Funding

This work was supported by the National Natural Science Foundation of China (NSFC31600380, 31701831, 42007049, and 31570429) and Henan Science and Technology Research Project of Henan Province, China (222102110126). Dr Chen is granted by EU H2020 Marie Skłodowska-Curie Actions (No. 839806), Aarhus University Research Foundation (AUFF-E-2019-7-1), Danish Independent Research Foundation (1127-00015B), and Nordic Committee of Agriculture and Food Research.

## Conflict of Interest

The authors declare that the research was conducted in the absence of any commercial or financial relationships that could be construed as a potential conflict of interest.

## Publisher's Note

All claims expressed in this article are solely those of the authors and do not necessarily represent those of their affiliated organizations, or those of the publisher, the editors and the reviewers. Any product that may be evaluated in this article, or claim that may be made by its manufacturer, is not guaranteed or endorsed by the publisher.
