# Supplementary material for: Belowground Root Competition Alters the Grass Seedling Establishment Response to Light by a Nitrogen Addition and Mowing Experiment in a Temperate Steppe
Source: Front Plant Sci. 2022 Jul 14;13:801343. doi: 10.3389/fpls.2022.801343 (PMC9331913; doi:10.3389/fpls.2022.801343)
Supplement: Supplementary file 1 [file Table_1.pdf]

Table S1

Results (F-values) of three-way ANOVA on the effects of species (SP), root isolation (RI), mowing (M)\nitrogen addition (N), and their interactions on total biomass, height, survival, shoot and root biomass, and root/shoot.

Source of Variation	Total biomass	Height	Survival	Shoot	Root	Root/shoot
SP	0.00	111.966***	0.22	1.194	4.484*	6.645*
RI	52.863***	43.233***	2.00	52.976***	40.331***	8.582**
M	0.02	0.01	3.56	0.002	0.208	0.819
SP* RI	0.00	1.28	0.89	1.226	5.549*	24.561***
SP*M	0.28	0.70	2.00	0.263	0.253	0.262
RI *M	0.00	0.05	0.22	0.003	0	1.852
SP*RI*M	0.14	0.82	3.56	0.131	0.133	0.26
SP	0.094	43.339***	0.067	0.65	1.068	0.016
RI	25.915***	17.136***	0.332	22.88***	27.328***	5.614*
N	20.083***	3.671	13.111**	16.971***	23.414***	0.417
SP* RI	0.028	2.049	0.242	0.595	2.034	6.409*
SP*N	0.05	2.958	0.114	0.675	1.839	3.441
RI *N	10.953**	2.324	0.246	9.342**	12.512**	1.035
SP*RI*N	0.058	0.06	1.384	0.678	1.707	1.511

Table S2

Results (F-values) of two-way ANOVA on the effects of root isolation (RI), mowing (M)\nitrogen addition (N), and their interactions on total biomass, height, survival, shoot and root biomass, and root/shoot of *S.krylovii* and *C.squarrosa*, respectively.

Source of Variation		Total biomass	Height	Survival	Shoot	Root	Root/shoot
S.krylovii	RI	31.515***	21.266***	2.97	40.545***	27.427***	0.589
	M	0.385	0.707	4.788*	8.104**	7.23**	0.164
	RI*M	0.034	1.14	1.152	4.188*	4.197*	0.005
	RI	22.753***	15.978**	0.364	26.086***	17.733***	0.155
	N	13.711**	3.231	9.091**	13.72**	13.039**	2.707
	RI*N	8.832**	2.524	0.364	8.163*	9.303**	2.194
C.squarrosa	RI	22.658***	11.582**	0.095	23.338***	18.084***	38.01***
	M	0.148	1.756	0.095	0.187	0.035	0.951
	RI*M	0.014	1.247	0.857	0.006	0.076	5.061*
	RI	8.611*	3.209	0.064	8.125*	10.461**	6.266*
	N	8.836**	0.065	4.589*	8.241*	11.215**	3.102
	RI*N	3.814	0.703	1.82	3.848	3.513	0.06
